# Ethnographic Film, Fourth Cinema and Situated Knowledge Practices—from Colonial Film to Collaborative Research

**DOI:** 10.1007/s00048-024-00399-w

**Published:** 2024-08-12

**Authors:** Julia Bee

**Affiliations:** 1 Ruhr-Universität Bochum, Bochum, Deutschland; 2 Universität Siegen, Siegen, Deutschland

**Keywords:** Collaborative filmmaking, Collaborative anthropologies, Ethnographic film, Situated knowledge, Feminist STS, Kollaboratives Filmemachen, Kollaborative Anthropologie, Ethnographischer Film, Situiertes Wissen, Feministische STS

## Abstract

The text attempts to understand the development of collaborative audiovisual knowledge practices in anthropology as situated and diffractive knowledge (Haraway, Barad, Smith). By considering specific stages in the history of collaborative and participatory projects, the article argues that collaborative filmmaking is not only a decentering of one-sided authorship and one-sided modes of representation, but also a media-specific form of knowledge that is bound to and embedded in social contexts. Through the example of colonial film, the article describes stations of demarcation and attempts to decolonize film. Current film experiments with marginalized groups have their origins in “shared anthropologies” (Rouch) and have further developed this approach through more consistent forms of Fourth Cinema and power sharing with Indigenous communities. Film is thus also able to depict amateur knowledge practices within collaborative research projects.

## Abstract (de)

Der Text schlägt vor, die Entwicklung kollaborativer audiovisueller Wissenspraktiken in der Anthropologie als situiertes und diffraktives Wissen zu verstehen (Haraway, Barad, Smith). Im Durchgang durch einige historische Stationen der Geschichte kollaborativer und partizipativer Projekte wird vorgeschlagen, dass kollaboratives Filmemachen nicht nur eine Dezentrierung einseitiger Autor:innenschaft und einseitiger Repräsentationsmodi ist, sondern auch eine medienspezifische Wissensform, die an soziale Kontexte gebunden und in diese eingebettet ist. Vor dem Hintergrund des Kolonialfilms werden Stationen der Abgrenzung und Versuche der Dekolonisierung des Films beschrieben. Aktuelle Filmexperimente mit marginalisierten Gruppen haben ihren Ursprung in den „shared anthropologies“ (Rouch) und haben diesen Ansatz durch konsequentere Formen des power sharing in Indigenen Kollaborationen und im Fourth Cinema weiterentwickelt. Film kann so auch Amateur:innenwissenspraktiken in kollaborativen Forschungsprojekten abbilden.

In this article, I discuss some of the ways in which filmmaking has been deployed for research in anthropological and activist contexts. Building on the history of ethnographic filmmaking, I understand collaborative filmmaking as a cooperative method that is media-specific and participatory, and generates specific forms of knowledge. This knowledge is situated, often locally based, and combines communitarian and social approaches to participation and shared authorship in filmmaking.

Collaborative filmmaking is not only filmmaking by a crew with several members; as a collective endeavor, it involves shared authorship and the shared development of a research question or goal—aesthetic, academic or embedded in other forms of (journalistic or reportage) knowledge production. Collaborative ethnographic filmmaking was seen as “decolonial option” (Albrecht & Walter 2019) for working with marginalized groups and individual collaborators.

It involves professional filmmakers (training that often overlaps with the position of an anthropologist) as well as academics and/or members of a community without formal training (in terms of filmmaking and/or academic access to education), bringing together different modes of formal and informal knowledge in service of a common project. In what follows, I discuss ideas related to this type of collaborative filmmaking, mostly stemming from visual anthropology and ethnographic filmmaking, including its pre-histories and influences, which often do not meet the exact criteria outlined above, but have historically fed into the idea of collaboration in ethnographic work and filmmaking beyond the mere notion of cooperation on the basis of informed consent in anthropology, and were aimed at leveling power relations.

Often collaborative ethnographic filmmaking involves sharing skills, equipment and funding. Collaborative filmmaking is thus potentially an epistemic and social intervention. Nonetheless, the focus on filmmaking does not mean that other forms of artistic and epistemic collaboration, including writing, photography or collage, that address the larger problematic of politics of representation in both authorship and media do not exist. While participatory projects of all sorts—from witness reports to reenactment in cinéma vérité to educational film projects—are an important part of the genealogy of ethnographic filmmaking, I am trying here to think through collaboration as a more consistent displacement of epistemic hierarchies by developing research questions within the specific medium of the audiovisual.

Documentary filmmaking has come to be understood as a collaborative tool for research and for community purposes (White 2003; Zoettl 2012; Liberman 2019; Schönhofer 2019; Gruber 2016), starting from the assumption that “film is not just a form of representation. […] Filmmaking itself is a form of social practice” (Albrecht & Walter 2019: 1). While film is often realized through the cooperation of a crew, collaborative filmmaking, also called participatory filmmaking,^1^ is based on both the training of amateurs at the camera for self-documentation and the decentering of the auteur-filmmaker in ethnographic or sociological film. Collaborative filmmaking—in a nutshell—means that people who were previously the object of filming or have had their lives “explained” by documentary filmmakers or anthropologists now become actors, witnesses, or experts in the filming of their perspectives (Rouch 2003: 46)—and thereby often become filmmakers, too. In the history of ethnographic collaborations, this has for example occurred in collaborations between Indigenous and non-Indigenous filmmakers (Ferreira Pinheiro & Ferreira Pará Yxapy 2019). This approach aims to shift problems of authorship and challenge existing power relationships in the representation of certain communities—but surely cannot solve all of them for good (Liberman 2019). Following Schiwy (2009), Graham (2014a; 2014b) and White (2003), media practices are thus seen to facilitate cultural mediation (Ginsburg 1995) for marginalized groups, as well as change symbolic power relations and redistribute the recognition for academic and artistic works (Schönhofer 2019).^2^

Although ethnographic accounts are usually based on cooperation in order to meet the ethical standards of ethnography, collaboration in ethnographic filmmaking occurs to different degrees, ranging from dramaturgical involvement, witness reports, and storytelling to film training, shared authorship, and a “handing over [of] the camera”.^3^ By following these variations in how anthropologists and filmmakers collaborate with experts of everyday life across different projects, it becomes possible to discuss collaboration as an epistemic intervention:^4^ Rather than seeing film as *either* a tool for community engagement *or* for fulfilling anthropological research goals, collaborative filmmaking should thus be understood as an activist research tool that simultaneously functions as a medium of knowledge production. In *Research as Resistance, Revisiting Critical, Indigenous and Anti-Oppressive Approaches* (2015), Susan Strega and Leslie Brown, among others, propose a conception of activist research in which anthropologists and social scientists—together with the communities they are working with—collaborate to develop research questions and methodologies that might be beneficial for those communities, rather than “extracting” knowledge from them (Kovach 2015: 32; Absolon & Willett 2015: 112; Strega 2015: 229; Potts/Brown: 274).^5^ In the realm of filmmaking, this means producing self-authored images, perspectives, and knowledge in concert with the communities involved in the film. Collaborative filmmaking is thus an embodied and practical form of knowledge production that aims to entangle the often contrasting fields of epistemology (knowing) and the social (being).

Although participation and collaboration have become increasingly important in anthropology (Boyer & Marcus 2020), collaborative filmmaking does not yet have a single conventionalized methodology, borrowing instead from different participatory and emancipatory traditions. Gruber (2016: 17) suggests following three lines of filmmaking to understand the impact of collaborative filmmaking: Indigenous media (Ginsburg 1995), which here will be referred to as fourth cinema (Barclay 2003a; Barclay 2003b); anthropological filmmaking; and participatory video. Gruber worked with collaborative and participatory methods in the Okavango Basin from 2011 to 2013 (*Huchi-Honey*, Antónia et al. 2013; *Liparu Lyetu—Our Life*, Gruber et al. 2011; *The Secret of Our Environment*, Gruber et al. 2013), co-producing three films with what he calls “local stakeholders” (Gruber 2016: 25). For Gruber, collaboration is to be understood as joint filming, editing and screening, wherein the impact lies in the community for whom he is doing the film, as an “involved audience”. This differs from most of the work of visual anthropologists, who often make films to be shown in classes, at film festivals, or to the academic community. In what follows, I contrast historical and contemporary films to make visible the heterogenous ideas about artistic and ethnographic collaboration in which recent collaborative filmmaking is situated. It should be noted that, despite the use of historical examples from various contexts, I am not aiming for a *complete* historiography here.

I draw on contemporary examples of ethnographic filmmaking, before moving on to historical examples that inspired collaborative approaches in order to read these through a feminist and postcolonial lens informed by Donna Haraway, Karen Barad, and Linda Tuhiwai Smith among others.^6^ In contrast to early ethnographic film, which produced a colonial gaze and was the source of colonial perspectives, reflexive and critical forms of conducting research with ethnographic films have emerged in recent decades that work against visual colonization, often also in projects that move beyond anthropology, for example into activism and social work.^7^

In what follows, I begin with a brief discussion of ethnographic filmmaking, a discipline from which many projects emerged, as a tool for knowledge production. Drawing on examples from different eras of the history of ethnographic film, I propose that we understand collaborative ethnographic filmmaking as encompassing both knowledge and social purposes within debates in feminist science and technology studies and within postcolonial approaches in anthropology, in order to challenge both the notion of knowledge as a resource or an object to be extracted from its social context and its ability to be neutrally documented on film.

## Between Contemporary Ethnographic Filmmaking and the Arts

This section provides a short introduction into recent forms of collaboration between art and anthropology (Schneider & Wright 2013), as well as a more historical account of the idea of collaboration. An important thread in the genealogy of collaborative filmmaking is intertwined with both anthropology and ethnographic filmmaking, which has long raised the question of the position of the knowledge that films produce. Do ethnographic films represent knowledge or *are* they a form of knowledge in and of themselves? How does this change our understanding of knowledge? As a media scholar, I am interested in this question against the backdrop of the recent popularity of documentary forms circulating in art contexts that often have ethnographic content or use methods of cinematic observation.

While ethnographic filmmaking was long seen solely as a tool for communicating anthropological knowledge, it also became influential in the field of art beginning in the 2000s with films—like *Leviathan* (Lucien Castaing-Taylor; Véréna Paravel 2012)—produced by the Harvard-based Sensory Ethnography Lab. In addition, ethnographic filmmaking has been informed by a longer discussion of film as a form of knowledge representation, distribution and production in anthropology and beyond (Taylor 1996), beginning with Margret Mead and Gregory Bateson’s films from their fieldwork in Bali in the 1930s (e.g. later made famous in *Dance and Trance in Bali*, 1951).

On the one hand, ethnographic films have repeatedly been described as art and as forms of research in the context of visual anthropology (Grimshaw & Ravetz 2005; Schneider & Wright 2013), especially since artistic installation formats are often exhibited in ethnographic museums, and ethnographic themes are included in art museums. Schmitz and Gupta (2012), for example, suggest that the films of the well-known filmmaker Robert Gardner are a form of artistic research, and therefore should be understood as anthropology *and* art. Gardner’s famous and controversial *Forest of Bliss* (1986) about Varanasi in India is one example of the aestheticization of anthropological insights through editing and visual metaphors (Ruby 1991). On the other hand, others, like anthropologist Karl Heider (1976: 5), see ethnographic filmmaking as a conveyor of ethnographic research oriented toward the methods and values of anthropology. While the first perspective sees film as an autonomous research tool in the genealogy of artistic research, the second regards film as a mere tool for the distribution of anthropological knowledge for educational purposes—to popularize it or to make it accessible to the public by audiovisual means. This was common, for example, in television productions from the 1950s onwards, which mixed travelogue and reportage, building on a history of colonial photography and other media that is still present (and popular) today in the productions of television channels such as *arte* in Germany and France. Ethnographic film as art on the one hand, and film as educational tool on the other, describe two different roles ascribed to ethnographic filmmaking. Collaborative films can thus be seen as art or as an “educational resource” (Alexander Street n.d.), but can also be seen as a way of producing social relations and subjectivities in the very act of knowledge production. I would like to develop this last point further by looking at forms of involvement and participation in film history as precursors to contemporary collaborations between art and anthropology. While the acceptance of film as a source of knowledge in anthropology has been controversial, collaboration can be seen as an even greater challenge to the development of new methodologies.

What specific forms of collaboration exist today? Without claiming to be exhaustive, I would like to present a few typical forms. One approach that emerged in the last ten years is that of well-known ethnographic filmmaker David MacDougall, who taught children in India to document their own lives (for example *Eleven in Delwara*, 2014). This approach uses training on camera to bring in the perspectives of children from different social classes. While MacDougall had previously worked in schools in India, here, he shifted the perspective from that of an adult and *white* anthropologist toward an intrinsically childlike viewpoint by handing over the camera. This approach thus foregrounds the knowledge and perspectives of the children, in contrast to the more common focus on and valorization of adults in ethnographic accounts. As such, both the adult and the *white* perspective of the anthropological filmmaker are potentially replaced, if only temporarily.^8^ Similarly, audiovisual methodologies that work with children’s points of view were already being developed in Alanis Obomsawin’s early body of work. In *Christmas at Moose Factory* (1971), for example, Obomsawin collaborated with Cree children by montaging their drawings and stories expressing their experiences of the Canadian residential school system in the 1960s. In *Christmas at Moose Factory,* it becomes clear how oral traditions of archiving history and visual methodologies like drawing, filming, and montaging music, speech, and sound can intersect.^9^ Film seems to be an appropriate tool to document these intersections and to create an assemblage of different audiovisual media.

In recent years, collaborative approaches have often led to the formation of film collectives (or vice versa: film collectives have been formed to experiment with collective authorship), as in the case of the Australian Karrabing Film Collective, which began in 2008. Karrabing developed an approach that combined science fiction and experimental filmmaking, wherein art and political activism were intertwined. Specifically, the dreamlike perspectives in *The Mermaids or Aiden in Wonderland* (2018), a film about fracking on Aborigine land, become a way to express the entangled cosmological and socio-psychological impact of ecological damage. The knowledge created here is closely linked to the aesthetics and form of the film; the surreal mode of using point-of-view, subjective and distorted perspectives, as well as sound and color filters help to articulate different realities at the same time. Karrabing creates visions and perspectives beyond the realism or even positivism of factual filmmaking, challenging strict documentary modes by deploying speculative fiction on the level of mise-en-scène and narration. Nevertheless, it is still a form of sharing knowledge about land grabbing and fracking, for example, in which psychosocial, spiritual and political dimensions are also inscribed. The form of the collective, which I will come back to in relation to the Americas, is a way of working in a self-determined and polyphonic way. It has largely emancipated itself from guidelines and claims to participation in favor of its own aesthetics and themes. Although the anthropologist Elizabeth Povinelli is involved in the collective, this is a film collective, and as such these are not anthropological films in the narrower sense. This does not mean, however, that the subject and the aesthetic approach of the films are not relevant to an examination of new forms of knowledge for anthropology.

Flight and the experience of racism are often central themes that motivate cooperation in the field of art and film. The 2016 collaborative film *Les Sauteurs* (Abou Bakar Sidibé, Moritz Siebert, Estephan Wagner) documents forced migration and life at the borders of Fortress Europe by giving a camera to Abou Sidibé, who now lives in Berlin and documented his flight together with the two filmmakers (Seibel 2019). Sidibé filmed his flight and stay in a self-organized camp at the border in the Spanish enclave of Melilla, documenting the border practice of pushbacks and the social structures in the camps through his eyes. Sidibé was also involved in the post-production and editing process. This approach is certainly one of the most consistent, combining self-representation with self-determined camera work and shared authorship.^10^ The film combines approaches that I will discuss below from a historical perspective, namely the work in front of *and* behind the camera as well as in the editing process. Here, the camera is handed over to break the cycle of racialized othering, especially in Germany after 2015 (where the film was produced), a year dominated by populist discourses on migration. The film is based on the experiences of those affected by flight and adds images produced through their own authorship—in contrast to the externally determined image discourse, thus centering their own priorities for the film. Nonetheless, this “insider’s view” does not always prevent an exoticizing and voyeuristic perspective in filmmaking in general. Although videos of other flights exist on social media, this 80-minute film format allows for a different form of circulation—in art contexts and film festivals like the Berlinale—and create a different dramaturgical narrative than do videos on Youtube.

These are typical forms of collaboration in the space between art, activism and anthropology. While MacDougall is a renowned anthropological filmmaker and Elizabeth Povinelli, a founding member of Karrabing, is a much-read anthropologist, the crew from *Les Sauteurs* hail from the fields of art and education. Nevertheless, each of these films was screened in art contexts and some at (ethnographic) film festivals targeting a larger audience beyond academia. Due to the mediality of film, the images also appeal to very different spectators beyond anthropology. Very few of these types of collaborative projects remain exclusively within the framework of ethnography, but instead transcend it through social work, participatory art projects and experimental film and video art.^11^ Ultimately, however, these changing claims to representation apply to the field of research as well as to the field of film.

## Early Ethnographic Filmmaking

But what do these approaches actually provide? In this section, I want to sketch out the contours of early ethnographic film as a colonial medium to illustrate the problem from which ethnographic film still has to emancipate itself today and to help explain what collaborative practices are responding to. Ann Kaplan (1997: 61), among others, has observed that film and colonialism developed concurrently around 1900: Film was integral to anthropology from the beginning, serving as a documentary tool for anthropological expeditions while at the same time showing up in popular cultural contexts such as fairs. Cameras were involved as early as the Torres-Strait-Expedition (1898–1899) and were used to document dances and rituals; photography (Edwards 1992) and filmmaking embodied anthropological assumptions immersed in nineteenth-century race theory (Fuhrmann 2009: 349 f.). Although film and photography were seen as mere documentation tools without aesthetic demands, they nonetheless embody the worldviews of the colonial technique; more so, they *operated* as the very techniques of colonialism in the framework of multi-disciplinary research travel. Film was also used to popularize anthropological knowledge and, vice versa, to make popular knowledge appear anthropological and therefore educational, especially later during the Weimar Republic through the educational format of *Kulturfilm*.

Films presented the perspective of the colonizers and documented their colonial projects, including missions (Meyer 2005) and the building of infrastructure. Early ethnographic films established viewing relations that not only embodied but also perpetuated, stabilized, and produced power relations between the colonizer and the colonized: they turned individuals into objects for the Western gaze (Hall 2004). The obsessive classification of humans and the fetish for filming Indigenous populations has often been criticized by film studies scholars (Hansen et al. 1989)—and in the films themselves (Trinh T. Min-ha, *Reassemblage* 1982). Wolfgang Fuhrmann (2009; 2015) describes early ethnographic filmmaking around 1900 as deeply involved in colonial structures of perception. Tobias Nagl (2009; 2018: 163) argues that these relations were, at least in the German context, revived in the Weimar Republic after Germany lost its colonies, as ethnographic films became ways to nostalgically remember what was “lost” and served to feed national and revanchist discourses and feelings. Both during Germany’s official and institutional colonial period and after losing its colonies, films created viewing relations that strengthened the bonds between colonial actors and European viewers by legitimizing the colonial project: In the style of travelogues, spectators were able to experience regions they perceived as belonging to Germany (or to other European countries) (Nagl 2009; Nagl 2018).

Films like Hans Schomburgk’s *Im deutschen Sudan* (1917) provided episodic insights into the landscapes and population of the (former) German colonies. This film observes and comments on Indigenous working and craft practices and can be seen as an appraisal of an unpaid working force. The film can be regarded not only as a portrait of a particular territory and its available resources, but as an advertisement for its work force (Königshofen 2020).^12^ Typical for earlier anthropological photography, the film displays anthropometric studies of individuals classified as part of certain classes in local society or characterized by what were viewed as typical occupations like “Bogenschütze” (bowman) (Nagl 2009: 254–260). In addition, intertitles “explained”, and therefore trained, the perception of viewers—namely viewing relations that objectify, measure, and classify othered communities through a typical and popularized anthropological gaze.^13^ In the Weimar Republic, ethnographic films like Schomburg’s were shown in cinemas as *Kulturfilme* (cultural films), that would educate spectators in different scientific and pseudo-scientific fields. They were meant to be educational, and successfully created a hierarchized gaze and sense of possessiveness toward the German colonies. Informed by early techniques of visual colonization like Schomburgk’s, later filmmaking has been forced to reckon with this problematic heritage. One of the important tasks of the documentary cinéma vérité in the 1960s, in the era of formal decolonization, was thus the *subjectivization* of protagonists in the (former) colonies, which challenged the *objectivization* produced by previous viewing relations. In the 1960s, as I will explain below, this was also attempted by refraining from talking about people or explaining images with a god-like voice-over, but instead creating modalities of direct speech through synchronous sound technologies.^14^

Longer narrative documentary films with ethnographic themes were first produced at the beginning of the 1920s. Among others, *Grass* (Merian C. Cooper/Ernst B. Schoedsack 1925) and *Nanook of the North* (Robert J. Flaherty 1922) created romanticized visions of colonial settings such as Oceania or, famously, the Canadian Arctic. Although different assessments of *Nanook of the North* exist, the film is widely understood as problematic for its romanticized and childlike representation of the Inuit characters. At the same time, the person renamed Nanook for the occasion by the filmmaker—his name was in fact Allakariallak—appears as a (partly individualized) hero in a “hostile” environment, an idealization of pre-modern life that valued Inuit cultural techniques while also romanticizing them. Despite the film’s many issues, its model of filmmaking, presumably involving Allakariallak in certain parts of the process—which can cautiously be assumed by the fact that Allakariallak looks into the camera, revealing his daily life (Perez 1998: 47)—inspired later filmmaking by Jean Rouch in the 1950s (Rouch 2003: 62). It can thus be seen as the basis of an admittedly limited collaborative project, working with amateur actors portraying their own lives. Rouch’s notion of “shared anthropology” (2003: 44) was informed by his positive evaluation of Robert J. Flaherty’s “participating camera” (Rouch 2003: 98–99; Seibel 2019) as a way of telling a story together instead of by just assembling material (Grierson 2012).

## Documentary Revolutions in the 1960s and 1970s: The Basis for Collaboration

Historically and conceptually, the important shifts on which contemporary collaborative experiments are based emerged in the early 1960s (Waugh et al. 2010; Crocker 2003), some in direct connection with the decolonial movements of Third Cinema, some later inspiring the new waves of European auteur cinema. In the Americas, Africa, and Asia, decolonial film movements formed with the goal of breaking through the Western gaze on the countries of the Global South (Solanas & Getino 2003). After First Cinema (Hollywood) and Second Cinema (European auteur)—Third Cinema wanted to create a decolonial film movement with noncommercial film circulation and production that emphasized collective labor and political decolonial agitation through film (Fernando Solanas, Octavio Getino: *La hora de los hornos* 1968). Following this, Barry Barclay (2003a; Barclay 2003b) also hoped to support Indigenous perspectives not represented in Third Cinema with the notion of Fourth Cinema, to which I will return below.

Both in the film industry and in sociology and anthropology, the documentary films of the 1960s and 1970s were understood to carry the potential for social change. Although citizen participation, as it has developed since the 1960s (Gruber 2016: 24), has had a major impact on the methods of collaborative filmmaking, the model of shared camerawork, editing and screening has not always been realized in full in each project. The Canadian program Challenge for Change/Société nouvelle, founded in 1967 and funded by the National Film Board of Canada (NFB), for example, deployed sociological perspectives aimed at the critical exploration of poverty and the class system (Druick 2010: 345). Through individual projects within this framework, the program provided citizens with the mass media training necessary to become journalists or ethnographers themselves and to be able to produce self-reflexive imagery and narration. Since 1968, for example, it has supported the collective The Indian Film Crew (sic!), with the goal of increasing the self-representation of First Nations filmmakers. The Indian Film Crew was not a collaboration between anthropologists and filmmakers, but between Indigenous filmmakers and institutions, and thereby adds a new layer to the history of collaborative filmmaking.

Challenge for Change combined education *through* film with education *in* filmmaking. It can thus be described as an early form of citizen science or citizen journalism, except that it was aimed at empowerment rather than knowledge production. Such approaches are rooted in the idea of creating a film with amateurs, of filming together to collect the perspective of those directly involved as a form of “activist documentary” (Waugh et al. 2010). As such, they encompass many of the ideas later picked up by other collaborative approaches. For example, Bonnie Sherr Klein worked with a francophone community in *VTR St-Jacques* (1969) on supporting their self-documentation and documentary expertise. A series of films by Colin Low made on Fogo Island in 1967 with local fishing communities became a model for film as a tool to document changing communities and to provide a means of exchange and connection with other communities through the production and reception of films at a time when the possibilities for such circulation were more limited than after the advent of social media. The process-based character of filmmaking thus became very important (White 2003; Crocker 2003): At Fogo, communication technologies like film facilitated the self-organization of the community (Crocker 2003), a phenomenon dubbed the “Fogo process” and discussed enthusiastically in the literature as an exemplar of the social facet of this type of filmmaking (White 2003; Crocker 2003). Nonetheless, this approach was not collaborative in the same way as, for example, Bonnie Sherr Klein’s media activist approach to training people on camera.

Ultimately, the first step was to produce images of everyday life under changing economic conditions and to allow for empathy and a sense of connection; the second was to have these images produced and promote broader participation through film workshops. In addition, through projects like The Indian Film Crew, a collective was itself directly supported. As such, Challenge for Change represents a certain culmination of the various means of participation or cooperation that developed over the years in different places around the world.

I would like to turn here to a film that—together with Jean Rouch’s films—fundamentally prepared the ground for working together with a community on their self-representation, thus becoming an important model for subsequent projects. *Pour la suite du monde* (Pierre Perrault, Michel Brault, Marcel Carrière 1963)—a film in the journalist ethnographic tradition, also produced by the National Film Bord of Canada—collaborated with a fishing community in Québec on their perspectives on whaling practices. Today, this film is part of Québec’s francophone heritage and contributed to the province’s Quiet Revolution, which brought about the awakening French-Canadian self-consciousness (Scheppler 2006). Perrault, Brault and Carrière used collaborative approaches to oral history to present the different perspectives within the community on a historical event that defined them culturally. Collaboration here entailed a dialogue-based and multi-perspective approach made possible through the medium of film—a medium that could then also be widely distributed. The three authors remained the main filmmakers, and Brault the camera man, but they put themselves at the service of the community, just as the community became the main actor in their film (Deleuze 1989). The position of these journalist-filmmakers corresponds most closely to the model of anthropologists as advocates of a community, but opens up this practice in two directions instead of depicting a one-sided representation of interests.

In *Pour la suite du monde*, Deleuze (1989: 150; 1990) sees a model for a new philosophy of filmmaking. Through their involvement in the filmmaking, the community members changed the way they communicated with each other and engaged with their own half-forgotten heritage (Scheppler 2006; Corneil 2010; Deleuze 1989). The occasion of the film inspired people to tell their stories and contribute to the archive of history and histories. Throughout, the film documents the process of reconstructing memory and reveals the protagonists’ emerging, different, often conflicting versions of their history. In the end, a long lost whaling practice re-emerges—albeit a new version, in which the whale survives.^15^ The Québecois dialect prevalent in the film was one of the most important elements of the valorization of French-Canadian culture. The process in which filmmaker Perrault, together with cameraman Michel Brault and sound artist Marcel Carrière, not only portrayed, but supported and introduced the cultural activities in the film became a model for Deleuze to conceptualize the interventionist practice of Perrault’s so-called “lived cinema” (cinéma vécu) as contributing to a paradigmatic shift in the history of documentary filmmaking.^16^ Especially the collective reenactment of history became a model for documentary films, wherein the combination of oral history and the re-invention of rituals by introducing a camera to record processes of reconstruction provides a model for collaboration that prepared the ground for other cultural-activist projects based on filmmaking. The collaboration of at least some (mostly male and white) islanders is crucial not because they give interviews and tell their version of the story, but because this collaborative perspective places the narrative itself at the center—the real subject thus becomes not the actual historical event, but the very *process* of (re)construction. Although the story seems to have been developed together or at least developed together during the process of filmmaking, authorship and aesthetic decisions nonetheless remain in the purview of the filmmakers.

In the last twenty years, initiatives have taken up similar questions arising from collaboration by democratizing film education by opening it up to marginalized groups and taking authorship a step further.^17^ Especially in terms of collaboration, the educational potential of film is thereby emphasized, in order to enable groups and actors to shape the field of filmmaking in new and different ways. Gruber (2016: 37) has stated that PV (participatory video) does not necessarily educate filmmakers, which can be seen in the different approach pioneered by Wapikoni Mobile, as an example of a combination of collaboration and more sustainable skills sharing and skills distribution. Traces of the 1960s, including the Challenge for Change program and in particular training filmmakers to create self-authored images, are still prominent in Wapikoni Mobile, which started in Canada in 2004 and promotes filmmaking workshops in different parts of the world today. The model for cooperation here is shaped by documentary platforms. In this case, the distribution is digital- and platform-based—which can potentially reach a wide audience, but may not automatically lead to a film screening in a local community. Wapikoni Mobile professionalized Fourth Cinema filmmaking by curating hundreds of semi-professional or amateur films by young adults. Mobile traveling video workshops offer the necessary infrastructure to First Nations and Inuit communities to make documentaries or video essays about their cultural practices and to facilitate professionalization by offering camera and editorial training. In so doing, Wapikoni Mobile facilitates semi-professional filmmaking, but also documents music, rituals, and memory, as well as contributing to a rich oral and audiovisual tradition by providing a publicly available media platform. Among the hundreds of short films, formats range from essay films to ethnographic style observational formats wherein children, youth, and the elderly provide their view on everyday life, art, crafts, tradition, transformation, identity, trauma, and cultural awareness. Wapikoni Mobile is thus not only a mobile film studio or a social media platform akin to Youtube or Vimeo (to which it is also connected), but curates these films within thematic categories like LGBTQI+ or two-spiritedness, among others. Through this process, Wapikoni Mobile not only documents and archives, but also contributes to First Nations and Inuit (film) culture and their critical assessments of settler-colonialism. It is an example of how different groups, generations, and artistic approaches contribute to an online film and knowledge culture based on artistic self-representation (Bee 2021). It is also a model of collaborative filmmaking, facilitated by film workshops, traveling mobile studios and a curated platform—while remaining within an educational framework, since it aims to train filmmakers. If the 1960s documentaries were characterized by synchronous sound and light cameras, today, digital platforms are inscribing new aesthetic forms into documentary filmmaking and creating new forms of reception and circulation. Although on-camera training already played a role in certain earlier projects, the collaboration model has now shifted toward the semi-professional training of the next generation of filmmakers, allowing for more sustainable collaborations between different participants in the program. This is particularly important, because in many countries there are formal and informal hurdles to accessing educational opportunities like film schools or university art programs.

As always in community filmmaking and film education, the question of methodology is crucial, since teaching film inherently communicates how film ‘works’ and thus directly influences how films are made, especially for younger participants. Film education and a culturally informed view on film became important, for example, in the 1966 collaborative project Navajo Film Themselves, in which Sol Worth, John Adair and Richard Chalfen attempted to research whether a Navajo film language was distinguishable from settler-colonial ones by handing out cameras to a Navajo community and analyzing their films (Navajo n.d.). Although this example is clearly one of the predecessors of Indigenous-settler film collaboration, it is characterized by the idea that Navajo films can be researched as an extension of Navajo culture itself. In this project, the central question thus became whether there was a universal or a specifically cultural film language. It was less a project of methodological decolonization—although clearly aimed at self-representation—than a sort of delegation of research questions to the participants (Mike Anderson, Al Clah, Susie Benally, Johnny Nelson, Mary Jane Tsosie, Maxine Tsosie, and Alta Kahn). Although these films are understood today as having created a self-representation that is different from the account produced by the anthropologists (or the communication scholars involved in this particular film project), it seems the films were not taken seriously by the researchers themselves as a form of knowledge production, but were seen as an experiment in researching the question of the universality or cultural specificity. The communication researchers concluded that the films had a different aesthetic in, for example, the mode of representing time, comparing them to elements of the nouvelle vague such as jump cuts. Instead of considering the specificity of Navajo storytelling in film, they compared it to the European avant-garde (Worth & Adair 1975). The films are nevertheless acknowledged as part of the culture and history of local communities, which have been rediscovered in recent years. Apparently, much more far-reaching symbolic techniques of collaboration, such as handing/taking over the camera and authorship, can lead to a situation in which the power relations associated with authorship and symbolic recognition are not necessarily destabilized, but highlighted. The interiority of everyday life and the self-documentation of the subjects is thus delegated to the work of the filmmaker.

In Europe, the United States and Canada, documentary filmmaking was radically changed by the advent of cinéma vérité, cinéma vécu and direct cinema at the beginning of the 1960s.^18^ As part of these movements, documentary film was no longer seen exclusively as a medium for conveying information, but could instead use fictionalization as well as spontaneous acting, which thus created the basis for collaborations in front and behind the camera. A film is no longer shot completely scripted and pre-authored, but is created at the same time as the scenes develop, allowing for a process of co-design. Here, I follow Gilles Deleuze (1989: 150–155; 1990), who suggested that documentary film in the 1960s shifted from documentation to intervention by using the speech acts of those formerly objectivized on camera in ethnographic filmmaking. Specifically in cinéma vérité, voice-over was no longer the only way to explain images; instead, actors could speak for themselves in *some* ethnographic films. New technical innovations also emerged that facilitated experimentation, such as synchronous sound and lightweight cameras.

The philosophy of filmmaking, which would become influential in shaping the role that filmmaking would later play in co-authorship, was not yet expressed in the sharing of directorial roles, but rather in the social and artistic intervention that a film aimed to achieve.

Jean Rouch, an engineer and filmmaker, for example, worked with young migrants in Ivory Coast in the 1950s and 1960s. He did not hand the camera over to his collaborators, but instead, as he says, gave “a voice to Africans themselves and asking them to comment directly on their behavior, actions, and reactions” (Rouch 2003: 62). He thus frames this experiment in inviting the protagonists to comment spontaneously on the images in *Moi un Noir* (1958) and *Jaguar* (1967, filmed in 1954) in post-production in the patriarchal manner of “giving” a voice.^19^ Later, Rouch provided support for workshops through Ateliers Varan, a training initiative for next-generation filmmakers that stemmed from his engagement in filmmaking. In *Moi, un Noir* and *Jaguar*, he often created spaces for participants to speak up or worked with re-enactments of peoples’ lives that crossed the boundary between fiction and reality, as in *La pyramide humaine* (1961). Here, again, self-authored or acted re-enactments are an important medium of collaboration, or, rather participation. With the exception of Ateliers Varan, the influence of cinéma vérité was for the most part not yet based on the method of “handing over the camera” as the important symbolic act of power sharing (Gruber 2016: 21; Seibel 2019), but focused instead on the idea of a documentary film as not only a representation of reality as it is through the lens of the filmmaker, but also as a form of intervention or experiment—a concept best embodied by films like *Moi un Noir, La pyramide humaine,* or *Chronique d’un été* (1961). Spontaneous, unscripted actions were seen as better way to access reality and a forum for participants to express themselves. Film thus becomes a medium of collaboration and provides a media-specific framework that not only has a greater reach than the anthropological monograph, but also contains an embodied, speech-act-bounded knowledge that enables other forms of collaboration beyond writing together. It is not only a source, but a specific media form, of knowledge transfer. Moreover, it also decenters powerful popular images by intervening in the sphere of the images themselves. As will be discussed in more theoretical detail below, it is a form of knowledge that is entangled with its medium.

Rouch understood film experiments as social experiments; in the first intertitles of * La pyramide humaine,* he expresses his view on film as a provocative “experiment”, asking Black and white students in Africa to become friends in late 1950s and early 1960s. The “provocation” (Feld 2003: 8) is in the very act of filmmaking, arranging a setting in which actors theatrically improvise or reenact their lives in front of the camera and introducing an element of play or newness. This makes it possible to leave the strict realm of documentary film in order to integrate fictional forms, such as participants using popular characters from film like secret agents or Tarzan to introduce themselves. The reality of what was formerly known as ethnographic documentary (and in parts of academia was to be understood as such for a long time) thereby became more complex, opening the idea of documentation to more experimental and even playful modes of filmmaking. Participants were given the opportunity to become, in part, amateur actors, as in *Moi un Noir*, in which they act out their own lives.^20^ Similarly, *Petit à petit* (Jean Rouch 1971) uses reverse ethnography in Paris, accompanying Lam Ibrahim Dia and Damouré Zika from Nigeria who—with a lot of humor and irony—research the Parisians (asking people on the streets for their size, mimicking the collection of anthropometrics). The participation of community members and the use of fiction has been termed ethnofiction and is used still today in various settings, from community films, like the recent *Not Another Vampire Movie* (fiktiv! 2021), to long-term documentary collaborative models, such as in *17 Blocks* (Davy Rothbart 2021).

By generating spaces for fictionalization or play, including the option to refer to oneself as someone else instead of representing reality as it is, film is often able to destabilize power systems like those found in films shot in colonies. Where early film aimed at fixing identities, Rouch, in some of his films, experimented with ways to set this “reality of the colonizer” into play, creating spaces for fiction and play as a specific framework of collaboration (Deleuze 1989: 150; Deleuze 1990: 125–26). Since the outcome of the improvised reenactment is not predetermined, it works with elements of risk and chance (Gruber 2016: 20, 34; Henley 2009), which are characteristic of ‘play’. The provocative camera does not falsify, but aims for greater truth, which is why Deleuze calls it the truth of cinema, in line with the notion of cinéma vérité. Rouch is not so much sharing his authorship, and thus his “symbolic power” (Schönhofer 2019), as he is contributing to the idea of film as a specific research medium that “provokes” reality.^21^

In terms of mixing fiction and reality, socialist and feminist movements also continued to influence collaborative styles in the 1960s and 1970s. Like Rouch and Morin, Cuban filmmaker and sociologist Sara Gómez used the sociological film interview with community participation in her work with amateur actors, self-representation through acting, and camera ethnography in everyday Afro-Cuban music culture. Gómez filmed with the perspective of a sociologist and, in particular, collected women’s perspectives on the socialist revolution, combining acting by nonprofessionals and professionals with interviews and her own account of machismo culture in *De cierta manera* (1973/74). In this film, she mixed reenactment, acting by amateurs from the community and educational sections with voice-overs. Sara Gómez’s films are not seen as canonical in the formation of cinéma vérité, although their unique mixture of historical and actual accounts, essayistic comments, and interviews with women, youth, and workers have produced a methodology of research as well as an intervention into the style of positivistic reportage films by using somewhat collaborative methods. Gómez was educated at the Cuban film school Instituto Cubano del Arte e Industria Cinematográficos and developed a unique socio-filmic methodology, creating an archive of different perspectives on post-revolutionary Cuba in her films and focusing on Afro-Cuban everyday life and culture, including music. Often, she herself or her voice appeared on camera while interviewing (*En la otra isla* 1968, *Mi aporte* 1972), which is different from, for example, Rouch’s approach (with the exception, for example, of his brief self-reflexive appearance with Morin at the end of *Chronique d’un été*).^22^ Moreover, Gómez’s work includes elements of 1970s feminist filmmaking, which created an audiovisual counter-archive of women’s experiences. Both Gómez’s works and films in the tradition of cinéma vérité engage with the notion that displaying undervalued everyday culture can be connected to the inclusion of protagonists who enact a story in the film that corresponds to their lives, but is not directly their own. Collaboration here is the joint re-telling of history on the basis of a narrative, fictionalized, story for educational and documentary purposes.

The collage of very different approaches presented in this section reveals how different political and aesthetic approaches to the problem of collaboration were developed in different places around the world in the 1960s and 1970s: socialist, postcolonial, feminist, and social democratic or new left approaches, all intended to address issues in film representation, ranging from racism to class society and sexism. Today, these different forms of citizen participation or collaboration in filmmaking continue to be used and adapted for film workshops around the world, maintaining the influence of collaborative, Indigenous, sociological, feminist filmmaking. I want to turn now to the Americas to focus on two initiatives of collaborative filmmaking: Vídeo nas Aldeias and Instituto Catitu, which produce films with Indigenous communities. Without wanting to tell an overly linear story, decade by decade, this section briefly traces how the collaborative anthropological and activist cinema of the 1980s took up the momentum from these earlier works.

## Collaborative Filmmaking in the Americas

In the 1980s, collaborative and activist films with Indigenous groups in Brazil began to engage with the practices of cinéma vérité in order to use more playful and spontaneous filming techniques (Graham 2014a; Graham 2014b; Aufderheide 2008). In a similar vein, Māori filmmaker Barry Barclay (2003a; 2003b) has formulated the contours of a Fourth Cinema movement driven by and for Indigenous communities, that facilitates authorship over images of oneself and allows for symbolic and economic recognition.^23^ The films in the following section borrow from both these ideas, often resulting in collaborative practices that stand between art, activism and anthropology.^24^ Particularly within anthropology, rethinking documentary practices has become a prominent concern, because images of people were often circulated without their consent, shown in ethnographic films, or exhibited in museums. In order to break the cycle of classification, objectification and exoticization, collaborative practices between Indigenous and non-Indigenous filmmakers have thus become particularly important (for example Caixeta de Queiroz & Diniz 2018). In recent years, the destruction of worlds through climate catastrophe, species extinction and ecocides has also encouraged the growing presence of Indigenous voices in audiovisual media, including Indigenous political and cultural activism on TikTok. But even before the advent of social media activism, forms of collaborative filmmaking were at the forefront of the creation of different accounts of not only Indigenous knowledge about ecological catastrophes, but also knowledge about resilience and resistance.^25^

Indigenous accounts of the climate catastrophe can be found in various films by Instituto Catitu, founded in 2009, with the aim “to contribute to the strengthening of Indigenous cultures and the defense of their rights through the use of new technologies as tools to express, transmit, share knowledges, from their own world views” (Instituto Catitu n.d.). The 2019 film *Heat* (Mari Corrêa 2019), for example, is a documentary made by those impacted directly by anthropogenic climate warming, filmed by anthropologist Mari Corrêa (founder of the Instituto Catitu), to share Indigenous knowledge about the climate catastrophe. Focusing on the experience of women, it brings together witness reports by female farmers, botanists, and micro gardeners about the local effects of catastrophic global warming. As the collaborative approach in this project is based on local knowledge and testimony, it is limited here to working in front of the camera.

The desire to create a platform for Indigenous knowledge practices through film can also change production structures and lead to collaboration in collective filmmaking initiatives. In their film *Inuit Knowledge and Climate Change* (2010), Zacharias Kunuk and Ian Mauro also focused on knowledge about ecological and climate catastrophes, building on Inuit reports and witnesses. While Corrêa, who does not identify as Indigenous, and Kunuk, a Fourth Cinema filmmaker, create oral and filmic archives and work with the perspectives of Indigenous communities to center their critical account of the climate catastrophe as well as the ecological measures implemented without their knowledge and participation, the direction of the collaboration often remains within the professional handling of the filmmakers. Kunuk, along with other filmmakers, co-founded the Isuma film collective, which promotes Inuit filmmaking based on the idea of collaboration within a professional framework, similar to Karraing in structure, but stylistically very different.^26^ The 2019 film *Ka’a Zar, Ukyze Wà* (*The Owners of the Forest in Danger*) by Flay Guajajara, Edivan dos Santos Guajajara, Erisvan Bone Guajajara, with the participation of Mari Corrêa, widened the concept of collaboration toward “taking over the camera” within the framework of the Instituto Catitu’s educational video workshop program. Supported by Instituto Catitu and Instituto Socioambiental among others, *Ka’a Zar, Ukyze Wà* (*The Owners of the Forest in Danger*) has a more collaborative approach and was directed by an Indigenous crew. In the film, members of the Guajajara community document members of the Awá Guajá community and thereby make clear through that very documentation that these groups do not want to be contacted. This seeming paradox underlines that (viewing) relations between different regional groups can be complicated, but also rooted in care for each other, as expressed in the documentation of a desire for “non-contact” as Marina Dias Weis has argued (Bee and Dias Weis 2019). While these films can be understood as activist filmmaking, they nevertheless produce knowledge and use collective as well as collaborative techniques.

Beginning in 1987, film workshops like those facilitated by Instituto Catitu and Wapikoni Mobile have also been developed by Vídeo nas Aldeias (Video in the Villages). In *From Ikpeng children to the world* (Natuyu Yuwipo Txicão, Karané Txicão, Kumaré Txicão 2001), for example, children introduce their village in the form of a video letter. Numerous filmmakers have collaborated with Vídeo nas Aldeias, including educators and filmmakers such as well-known filmmaker Divino Tserewahú, creating a space between collaborative film and Fourth Cinema.

Self-authored films by different NGOs working with Indigenous collaborators are deployed to contradict popular stereotypes, archive culture, communicate with other groups, and organize protests. Films have also be used to establish contact to other Indigenous groups or present oneself to them (Graham 2014b: 87), which Graham, following Faye Ginsburg, calls the “cultural activism” of Vídeo nas Aldeias. From the mid-1990s onwards, Vídeo nas Aldeias began working with techniques from cinéma vérité (Vídeo nas Aldeias 2014).

In particular, spontaneous everyday actions and playful performances with and for the camera were now in the foreground. In *Bicicletas de Nhanderú* (Patricia Ferreira Keretxu, Ariel Duarte Ortega 2011), for example, children reenact Michael Jackson’s “Beat it” with a dance transgressing pure documentation with playful segments using techniques established by cinéma vérité (Graham 2014b: 88). The film reveals different dimensions of experience and knowledge, including an introduction to cosmology, the display of theatrical elements, and moments of self-representation. These are specific forms of knowledge that combine embodied everyday experiences and self-identified knowledge with the possibility for viewers to encounter individuals who, through their bodily movements and speech acts relate knowledge to concrete experience and, simultaneously, translate and produce knowledge through collaboration.

As Freya Schiwy (2009) has argued, film has been used in the Americas for decades to communicate between villages, to represent oneself and thus to gain self-determination over one’s own image. Film thus becomes an important means of communication with the public, which is to be informed about land theft and unpunished killings. In addition, films counter stereotypes and are means of engaging with film culture (John 2019: 4; Graham 2014b: 88), producing a record of cultural practices, and actively participating in creating a dynamic and changing heritage (Lacerda 2018). Moreover, cinematic forms of communication can work as tools for coalition building and fundraising in, for example, the Amazon region and Mato Grosso in northern Brazil, where video activism such as Vídeo nas Aldeias and Instituto Catitu operate, and to communicate with other groups (Ferreira Pinheiro & Ferreira Pará Yxapy 2019: 7–8). This makes it all the more important to involve the groups and individuals directly affected in processes of academic and public representation—for which film can provide an accessible framework.

During this process of film development, a new form of knowledge—beyond the confines of anthropology—began to emerge, shaped by the different approaches involved. Although it began at the initiative of anthropologists and village communities, the aims of Vídeo nas Aldeias are now manifold. The films are not only aimed at non-Indigenous American and European audiences, but also other Indigenous groups: “Indigenous media creates networks through which Indigenous communities exchange information and thus create the possibility of developing alternative forms of political and economic organization” (Schiwy 2009: 57). That is an important shift away from self-directed ethnography, participation in films with an prepared concept, or academic research projects that explore the medium of film in individual or collaborative ways in the service of one’s own concerns or organization. In this new space, collaboration is possible: for example, through a dialogue-based form of mutual filming in the form of reciprocal video recordings that document and create a relationship, as in the work of white filmmaker Sophia Ferreira Pinheiro and Mbyá-Guarani filmmaker Patrícia Ferreira Pará Yxapy (2019).

Film, with its narrative, image, montage, and sound-based qualities can be a medium for negotiating issues of representation, and for reflexively asking about one’s own culture: what is important in filming, what is made visible or invisible, and who has the right to film and thereby represent a community or place? Following Faye Ginsburg’s (1995) notion of mediation of culture, the anthropologist Terence Turner speaks of “self-dramatization” in his work with Kayapo.^27^ In addition to anthropological research on collaborative filmmaking, these reflexive questions about filmmaking are also verbalized in films such as *A gente luta mas come fruta/We struggle but we eat fruit* (Wewito Piyãko, Isaac Pinhanta 2006), or in films that refer to the role of images in filmmaking, as in *I’ve Already Become an Image* (Zezinho Yube 2008).

In collaborative filmmaking, film can also participate in the interplay between socially, culturally, and ecologically sustainable practices, and therefore in fostering knowledge practices. The film *A gente luta mas come fruta*, for example, documents cultivation practices of forest and soil by a cooperative, and thereby forms a socio-ecological knowledge archive. Through the film, it becomes clear how much work is involved in the maintenance of a forest and what role film can play in the cultivation of an ecology—as educational and archival media. Knowledge, such as how seedlings are conserved or how water turtles are raised, is captured in *A gente luta mas come fruta* as an educational practice for the next generation and for other local groups. In the process of itself becoming a part of a regional knowledge culture, film can influence cultural, collective, and individual self-relationships, as well as relationships with other groups with whom it is important to share knowledge. In this way, the archive of knowledge and cultural techniques created by film is an archive in transition (Lacerda 2018), in so far as it also encompasses the struggle to form that archive and the feedback loop of those involved, which often comes in tandem with a non-academic knowledge culture. In films that provide grassroots knowledge production, like *We struggle but we eat fruit*, one can thus see two related processes: an ecological archive of techniques and practices of fostering water turtles and foresting seedlings—and the way the film as a cultural technique triggers processes in the community involved by including material screened in the film itself.

The 2007 Instituto Catitu production *Pïrinop, my first contact* (Mari Corrêa, Karané Txicão 2007) is an intervention in historic knowledge and anthropological archives with the help of collaborative film practices. The past is re-enacted from different points of view, similar to *Pour la suite du monde*, complementing and challenging the recordings produced by Cláudio and Leonardo Villas-Bôas, who resettled the Ikpeng group in 1964 to provide them with a territory. The technique of reenactment, inspired by cinéma vérité, is deployed to creatively challenge anthropology’s methodologies of knowledge production through the use of storytelling. Here, film acts as a trigger for processes that reveal not only the performative nature of memory and culture, but also how genuinely film is entangled with techniques and practices of memory, appropriation, and bricolage by using reenactment and witness reports as well as shared authorship. Described by Deleuze as an achievement of the 1960s, this makes the process-based medium of film interesting for the screening of cultural *processes* (instead of cultural states): Film records the act of creating culture and thus participates in that act rather than merely representing it. For example, negotiations over the process of reconstructing the first contact for a film show similarities in the negotiations that occurred in the 1963 film *Pour la suite du monde*. Here one can see forms of reenactment as well as dialogic parts that had been developed in earlier film collaborations. The film *Pïrinop* shows how the Ikpeng group wants to return to their original territories. The act of returning home becomes a task of reconstructing knowledge and resettlement for the group. Film, here, becomes a tool, a mediator, for collective processes of counter-mapping (Deleuze 1990). Collaborative and/or Fourth Cinema film can form a process-based archive based on film, becoming part of heritage in the making (Lacerda 2018). These forms of collaboration are thus not a representation from the outside, although they aim to represent and document lives, places and cultural processes for those on the ‘outside’. This is different from the preservation of culture that was accomplished in the early days of anthropology, including through film and photography, and often expressed as a gesture of ‘saving’ cultures—a rather euphemistic expression, as if groups were passively ‘losing’ cultural ties such as language, practices and cultural techniques.

Today, Indigenous knowledge is seen more and more as a resource against local consequences of the climate catastrophe, for example in gardening, building sustainable infrastructure, and aqua culture (Watson 2019). Learning from Indigenous knowledge, once a way to critically deal with the colonial devaluation of Indigenous heritage and traditions, now has the potential to become a new source for exploitation and appropriation (Smith et al. 2016: 141). But collective endeavors to archive knowledge can also mix different design approaches and help to develop ecological protection measures based on local knowledge, without essentializing Indigenous knowledge production. Approaches to combining different technologies and cultural techniques are performed in different parts of the world by NGOs like Rainforest Connections, which aims to prevent illegal logging with mobile phone detectors placed inside forests. In this way, film practices can be expanded to include other data-based collaborative design practices. Local forms of knowledge production and distribution through the medium of films can help to document and protest ecocides and environmental racism, as in Vincent Carellis, Tatiana Almeida, and Ernesto de Carvalho’s *Martírio* (2016): By collaborating with Indigenous groups in Brazil, Carelli (the founder of Vídeo nas Aldeias), Almeida and de Carvalho reveal the deadly connection between agro-industries, anti-Indigenous racism, and rural lobby politics in Mato Grosso do Sul. More so than it is the case in the above-mentioned films, he also provides a historical introduction to colonial land grabbing by using classic voice-overs.^28^

Each of these different films in the realm between collaborative film and Fourth cinema engages in different strategies and degrees of collaboration, ranging from participation, multi-perspective storytelling and witness reports, to shared camera work and filming and the establishment of cooperatives, all of which introduce different entanglements of knowledge and being, as I will explain in the final part of this text: *Heat* and *Martírio* collect witness reports, while *Pïrinop* is a collaboration between an anthropologist and filmmaker with the Ikpeng community; *We struggle but we eat fruit* is a set of self-filmed archival practices that document ecological practices and knowledge. They embody different degrees of collaboration in terms of their production, authorship, and distribution of knowledge, mixed partly with classical forms, such as the voice-over (*Martírio*).

Through their experimentation with aesthetics, films not only create novel forms of distribution of existing knowledge but intervene in the contexts in which they are created by providing new and more multifaceted perspectives than do academic texts—not only other stories, but other forms of storytelling, narration and representation of time (Fabian 2014). Freya Schiwy describes Indigenous filmmaking as a potential epistemic intervention (2009: 59), and in so doing takes into account not only the differences in content of different aesthetic and narrative approaches, but also the different possibilities of expression. The knowledge produced in Fourth Cinema and through collaborative filmmaking can thus serve communities, and is not knowledge about them, but extracted knowledge (Kovach 2015: 32; Absolon & Willett 2015: 112; Strega 2015: 229). This does not imply an instrumentalization of research for social purposes but rather an acknowledgment of the cultural, regional, and situated practices of non-academic research in which protagonists collaborate on the what and how of knowledge production by using the procedural form of collaboration in film production.^29^

Across contexts and decades, filmmaking itself has become a medium for social purposes, valuing the process as much as the product (Bessire 2009, 101; White 2003, 64–66; Zoettl 2012; Yúdice 2013: 252).^30^ This means that knowledge aspects not only become visible in the finished product, but also in the ways in which knowledge is created by using or inspiring techniques of community collaboration. Film, here, figures both as a research methodology and as a tool for community building (Crocker 2003: 132). Both operations intersect in the production, distribution, reception, and archiving of film that potentially feeds back into the communities from which it stems. In so doing, filmmaking produces knowledge as well as other social or psychosocial relationships (White 2003; Crocker 2003). Although practices of knowledge production exist in informal educational sectors in different regions, education is not something participatory filmmakers do as an unpaid service for the public; it usually aides their political or social struggle—which needs to be reflected and considered when building a methodology. The practices, economics and politics of each and every project thus need to be evaluated individually.

## Philosophies of Collaboration

I have presented several techniques that seek to intrude into the various power relations between academic filmmakers, often from anthropology and sociology, and the people they used to do research ‘about’, including colonial, adult-child, professional-non-professional, gendered or class relationships. These hierarchized categories are certainly often intersectional, for example when it comes to situationally destabilizing hierarchies between adult and child perspectives. Today, collaborative filmmaking may seem outdated, as social media enables a largely smartphone-based, accessible, video and selfie culture of self-representation. At least two aspects are, however, not obsolete: curating within subject areas that arise between the culture and social environment of those involved and the academic environment of the university or the curational environment of the museum—in the field of anthropology, for example; funding and payment for work are also important issues for the realization of more professional films. Self-determined representation can play a role here, in addition to jointly developed approaches to a knowledge practice. Furthermore, the concrete form of any professionalization is also key. For example, Wapikoni Mobile trains people in camera and sound work, in order to cover a whole range of expressive forms beyond social media aesthetics to support emerging forms of Fourth Cinema and to facilitate a new generation of filmmakers. Access to professional knowledge and technology is key here. Social media can change the curation and circulation of film, and its forms have certainly been inscribed in the aesthetics of film for some time. However, social media cannot replace filmmaking in its entirety, especially due to the length, narrative possibilities, mise-en-scène, editing and camera perspectives that enable a professional environment of production and post-production.

Although I have described numerous methods of participation, the question in terms of activist research is whether research questions were also developed together. This would mark a further step from participation to collaboration and would not only mean space for acting, speech acts and personal expertise, but would also have very concrete influences on the research design. While participation ranges from participation as experts of everyday life and actors in one’s own life to “handing/taking over the camera” or skill-sharing in filming, sound, editing and post-production, in contrast, collaboration describes the approach within activist research of jointly developing a research goal and a question (Strega and Brown 2015; Strega 2015; Kovach 2015). This is not to say that other participatory approaches do not have any added value. Participation can, for example, lead to further professionalization and to self-determined cinematic knowledge practices. Nonetheless, participation simply cannot prevent knowledge from being extracted from groups and places as stringently as joint research endeavors potentially can.

Wapikoni Mobile, for example, supports work on a filmmaker’s own chosen topic, even if they are not intended for research. However, like all images, the final product can be researched and thus turned into an object again, as can be seen in the history of material art in anthropology, especially since curated video platforms like Wapikoni Mobile circulate videos beyond the academic sphere. Self-determination and professionalization have thus developed beyond the collaboration with educational initiatives such as Wapikoni Mobile or Vídeo nas Aldeias, and have thereby created a visible public effect, as collectives have formed and filmmakers from groups that have traditionally been the subject of anthropology move from object of research to actors in their own right. These ‘postethnographic’ forms—post because they no longer exist exclusively under the purview of larger institutions or to meet conventional professional demands—add new situated forms of knowledge. I would therefore like to conclude with a theoretical discussion about the extent to which this knowledge is situated and how it intertwines content with the social. It is not only film that represents a particular form of knowledge, but also the practices of collaboration that shape its themes and aesthetics. This should be connected more directly, on the one hand, to the experiences of the participants and their views on film productions, and, on the other hand, to a more detailed analysis of concrete film productions.

## From Situated Knowledge to the Entanglement of Knowledge and Being

In the films described here, a knowledge intertwined with film emerges, a knowledge that narrates through images, bodies, and multi-sensuality (Pink 2015), of actors through speech acts, attitudes, etc.—what MacDougall (1999) calls the social aesthetics of a place (see also Born et al. 2017). This is one facet of situated knowledge, in which the media of knowledge production and knowledge are intertwined; another is the social configuration of knowledge, the social embedding in certain groups and the joint development of both research questions and possibilities of expression and representation. The collaborative practice of filmmaking can make such knowledge possible and provide space for it (Ferreira Pinheiro and Ferreira Pará Yxapy 2019). Conversely, the problems described in the first part on colonial filmmaking are also at risk of being repeated.

Donna Haraway describes ways of knowledge production that acknowledge partial views, taking into account their place of origin and the fact that they do not emerge from a vacuum (Haraway 1988). Building on Haraway, Karen Barad suggests a more radical view on the nature of situated knowledge. While Haraway proposes different ways of knowledge production as different epistemological approaches, Barad (2007: 83) suggests that different apparatuses produce entirely different “phenomena,” which she terms “ethico-onto-epistemologies,” drawing together “knowing and being” (2007: 185). Apparatuses, following Karen Barad, are practices of measurement that have a performative role in the research process (Barad 2007: 141–146). Barad, who has a background in quantum physics, describes the entanglement of apparatus and experimental research in generating a specific knowledge in which the apparatus of measurement inscribes itself. Following the wave particle dualism debate between Bohr and Heisenberg, Barad aligns with Bohr, arguing that the impact of apparatuses of measurement is not epistemological, but has ontological consequences. Reality does not simply withdraw its true nature from practices of measurement, but these practices create different realities depending on the performance of the apparatus of measurement—for example in the double slit experiment, wherein the representation of the wave- or particle-based nature of light depends on the measuring devices. In a very similar fashion, this phenomenon can be seen in ethnographic filmmaking. As ethnographic filmmaker David MacDougall (1998: 246) suggested, films and texts produce different kinds of knowledge depending on the materiality of the respective medium. This is especially true for ethnographic film, where different modes (styles, genres, etc.) of approaching reality and different filmic techniques articulate different realities—for example: Who is involved? Is a place understood through human or nonhuman actors and their agency? Is a place portrayed by narration, information, or experience? When comparing the documentary apparatus to the double slit experiment, ethnographic collaborative films thus generate specific entanglements of knowledge and being.

Although the apparatus of film is very different from those of quantum physics, the materiality of film can be conceptualized as interfering with reality, as producing diffraction patterns in Barad’s sense (Mohn 2023; in relation to other visual media: Bee 2018). This is reflected in the individual film’s aesthetics as well as in film production as a whole.^31^ The film apparatus has its own materiality and agency, and is not a mere reflection of an objective and fixed existence—an conception that has been discussed for decades in documentary filmmaking (but using different terminology). The onto-epistemological perspective, here, strengthens film as an important element in knowledge production, using it not to produce less, but rather *more* involvement.^32^ This approach asks us to consider the ways in which the representation of reality is not only negatively influenced or “polluted” by the social or the materiality of the medium in research processes, but also how the research processes, apparatuses, and the social world shape each other.

The apparatus here is not only concerned with technical or aesthetic aspects, but creates a specific socio-technical configuration, not only of film, but of collaborative practices between fields, generations, social positions and professions. Similarly, within the framework of decolonial methodologies, Linda Tuhiwai Smith et al. (2016) suggest understanding knowledge and being as entangled.^33^ Through the Māori concept of *IK mātauranga*, they build upon the idea that science often cuts through “knowing and being, grounded in traditional values, practices and philosophies, but [is] also constantly adaptable and made relevant to the world we live in” (Smith et al. 2016: 145). “IK mātauranga can and does co-exist with community-based expertise” (146). In contrast to essentialist ideas about authenticity and the eternal unchanging knowledge of Indigenous communities, the entanglement of knowledge and being here is used as a way to conceptualize procedural activist and collaborative research practices.

Knowledge produced by and for communities changes the nature of what is perceived as knowledge—namely that which is abstracted from that very context and community. According to Strega and Brown (2015), this applies not only to the definition of knowledge within academia, but touches on a wider context, namely the question of for and with whom knowledge is produced, and with what relevance. While Strega and Brown (2015; Strega 2015), Kovach (2015), Absolon & Willett (2015) among others (Alonso Bejarano et al. 2019), criticize the extractivist nature of knowledge production in working, for example, with Indigenous or other racialized communities, Smith et al. focus on the same problem that Barad proposes in quantum physics, but focusing more on the social aspect of the “apparatus”, as it were. For Smith et al., it is a question of not erasing the connection between knowing and being, seeing both closely connected and related to place and time. Film potentially brings these contexts back together, embodies possibilities of low-threshold and accessible collaboration that can connect body, language, knowledge and experience (Taylor 1996), and intervenes in logics of recognition (Schönhofer 2019). Although the contexts are quite different, the general argument is similar: By doing research, researcher-filmmakers produce not only knowledge, but also ways of being in the world for those involved, including the spectator who experiences different perspectives (MacDougall 1998) and the community represented—though all these relationships need to be better understood by looking closely at the practices and modes of individual projects.

Following Barad and Smith et al., knowledge can never be fully purified of social relations, as apparatuses are never disentangled from the materialities they are supposed to articulate. On the contrary, forms of artistic/filmic abstraction of knowledge do not necessarily mean the purification of knowledge, but can involve forms of engaging with the social context as methodology.

Potentially, some of the approaches to filmmaking outlined above embody situated knowledge reflecting the specific social contexts in which knowledge is produced. More strongly put, speaking through Barad and Smith et al., film intervenes in reality instead of reproducing it and thereby carries the potential to support social processes. All three processes described in this article are entangled and inform each other: knowledge production, filmmaking, and social processes as part of a community-based approach. The “apparatus” (Barad) of knowledge production is thus shaped by social and media practices, not by physically measurements. This perspective can be seen as an intervention not only in valuing and using documentary and ethnographic films as sources of knowledge, but also in seeing film as both a methodology of collaboration and, conversely, as a collaboration that enables knowledge practices. Understanding filmmaking as a collaboratively produced knowledge source can thus include many people and literacies.

While I have not evaluated each and every project in depth here, this article has sketched out a field with different approaches from different regions that influenced ideas about collaboration, some of which must be seen somewhat critically, despite their desire for power sharing. It is also important to continue the process of (self-)critiquing historical and recent approaches to collaboration and participation between anthropologists, filmmakers and activists, and to refrain from romanticizing them or perceiving them as the solution all to all issues of power. Rather, it can be said that collaboration opened up spaces for negotiating power relations, thereby beginning the problematization of the issue through newly audible voices in a specific framework created by filmmaking techniques.

## Filmography

- *17 Blocks* (Davy Rothbart 2021)
- *A gente luta mas come fruta [We struggle but we eat fruit]* (Wewito Piyãko, Isaac Pinhanta 2006)
- *Bicicletas de Nhanderú* (Patricia Ferreira Keretxu, Ariel Duarte Ortega 2011)
- *Christmas at Moose Factory* (Alanis Obomsawin 1971)
- *Chronique d’un été* (Jean Rouch 1961)
- *Cycles* (Zeinabu irene Davis 1989)
- *De cierta manera* (Sara Gómez 1973/74)
- *El coraje de pueblo* (Jorge Sanjinés 1971)
- *Eleven in Delwara* (David MacDougall 2014)
- *En la otra isla* (Sara Gómez 1968)
- *Forest of Bliss* (Robert Gardner 1986)
- *From Ikpeng children to the world* (Natuyu Yuwipo Txicão, Karané Txicão, Kumaré Txicão 2001)
- *Grass* (Merian C. Cooper and Ernst B. Schoedsack 1925)
- *Heat* (Mari Corrêa 2019)
- *Huchi-Honey* (Adelina Antónia; Martin Gruber; Miguel Sachilulo Hilario; Bino Henriques Job; Fatima José; Evaristo Quintas, 2013)
- *Im deutschen Sudan* (Hans Schomburgk 1917)
- *Inuit Knowledge and Climate Change* (Zacharias Kunuk and Ian Mauro 2010)
- *I’ve Already Become an Image* (Zezinho Yube 2008)
- *Jaguar* (Jean Rouch 1967, filmed 1954)
- *Ka’a Zar, Ukyze Wà [The Owners of the Forest in Danger]* (Flay Guajajara, Edivan dos Santos Guajajara and Erisvan Bone Guajajara 2019)
- *La hora de los hornos* (Fernando Solanas and Octavio Getino 1968)
- *La pyramide humaine* (Jean Rouch 1961)
- *Les Sauteurs* (Abou Bakar Sidibé, Moritz Siebert and Estephan Wagner 2016)
- *Leviathan* (Lucien Castaing-Taylor and Véréna Paravel 2012)
- *Lipara Lyetu—Our Life* (Martin Gruber; Clementine Shimbumburu Hamutenya; Tobias K. Immanuel; Veronica Ngundi Kapumburu; Samwel T. Kwandu; Johannes Mashare; Robert Mukuya; Angeline K. Simata; Raphael Sinkumba; Christofine Zangata 2011)
- *Martírio* (Vincent Carelli, Tatiana Almeida and Ernesto de Carvalho 2016)
- *Mi aporte* (Sara Gómez 1972)
- *Moi, un Noir* (Jean Rouch 1958)
- *Nanook of the North* (Robert J. Flaherty 1922)
- *Navajo Film Themselves* (Sol Worth et al. 1966)
- *Not another vampire movie* (fiktiv! 2021)
- *Petit à petit* (Jean Rouch 1971)
- *Pïrinop, my first contact* (Mari Corrêa, Karané Txicão 2007)
- *Pour la suite du monde* (Pierre Perrault, Michel Brault and Marcel Carrière 1963)
- *Reassemblage* (Trinh T. Min-ha 1982)
- *The Children of Fogo Island* (Colin Low 1967)
- *The Mermaids or Aiden in Wonderland* (Karrabing Film Collective 2018)
- *The Secret of our Environment* (Martin Gruber; Meshak Kwamovo; Gomotsegang Marungu; Chungudika Moeze; Rombe Mofundikwa; Keoratile Moruti; Mozumbi Mozumbi; Gongwe Wamana 2013)
- *The Single Parent Family: Images in Black* (Stormé Bright Sweet 1977)
- *Un film dramatique* (Eric Baudelaire 2019)
- *VTR St-Jacques* (Bonnie Sherr Klein 1969)
- *Whale Rider* (Niki Caro 2002)

## Footnotes

1 I avoid the term participation and use collaboration instead to make it clear that it is not about taking part in but about working together.

2 Collaboration is not only used to change modalities of filmmaking, but also to transform social processes (Malherbe & Everitt-Penhale 2017).

3 The phrase “handing over the camera”, which Gruber uses (2016: 21) for example, does not mean that Indigenous filmmakers have not actively asked for a camera. Later in this text, I will push this phrase into a more active position. Originally, Rouch argued that cameras should “pass into the hands of those, who, until now, have always been in front of the lens” (2003: 46).

4 Albrecht and Walter (2019) call this “epistemic disobedience”, borrowed from Walter Mignolo (2010).

5 Potts and Brown ask in relation to Indigenous communities: “What (whose) purpose does the research serve?” (2015: 274).

6 In the history of postcolonial and feminist critiques of ethnographic filmmaking, the films and texts of Trinh T. Minh-ha (1993) and others were important for reflecting on ethnographic filmmaking as a colonial knowledge practice.

7 For example, the project *Epopée* (n.d., since 2005), which produces self-directed and collaborative films with sex workers in Montréal, uses film as tool for community building. https://www.epopee.me/index-english-version.html.

8 One of the dangers of this approach is the assumption that footage created by those who are supposed to be the subject of an ethnographic film will produce a completely unbiased or authentic viewpoint. This question can also be asked in relation to other projects, such as the *Navajo Film Themselves* series, discussed later in this article.

9 For collaborations with children and youth, see *Un film dramatique* by Eric Baudelaire (2019), a project with children and teenagers that documented their lives and coming of age in a film club over several years.

10 Liberman (2019) not only handed over the camera, but also became the object of the collaborative filmmaker with whom Liberman works, an illegalized migrant called Mr. X.

11 Many of the filmmaking methods involved were however not developed out of a genuine ethnographic interest. They should therefore be called (post-)ethnographic in the strict sense to avoid self-determined productions being understood as mere auto-ethnographies.

12 I also mention this here because Schomburgk is still curated uncritically today, and documentary films are dedicated to his work as a so-called “expert on Africa”.

13 Tobias Nagl (2009: 255) describes this film as a hybrid between earlier episodic and later long films with stronger narration. Schomburgk was a traveler and safari specialist who also brought African animals to German zoos. He became well-known for his films on Africa, which he presented in colonial clubs and cinemas. His films continue to be shown as part of retrospectives even today, and his name is enshrined in the name of a neighborhood in the city of Querfurt.

14 Along this line of thought, Pat Aufderheide (2008) describes Jean Rouch’s approach as “strategies of subjectivization”.

15 Marit Kathryn Corneil (2010) termed this process of community history writing a “self-mythologization” of the francophone community. She describes the reconstruction of memory as a heterogenous and dialogical interplay of voices. Critically, one has to add, members of First Nations were not involved in this reconstruction of memory and are hardly acknowledged in the film’s re-writing of the (oral) histories of the island and of whale-hunting in particular. In addition, women are hardly ever heard on camera or involved in the hunting rituals.

16 The film has an ambivalent relationship to the French-Canadian settler-colonial past. The protagonists see themselves as the first settlers, although even in the film the Indigenous—the real First Nations—community’s presence in the consolidation of the first colonies cannot be denied (yet is given no voice in the film).

17 This is particularly the case in the area of cooperation with Indigenous groups. Approaches such as those of the Bolivian Ukamau group have dealt with the translation of Indigenous aesthetics and themes in film, albeit under the leadership of non-Indigenous actors. See *El coraje de pueblo*, Jorge Sanjinés 1971.

18 At the same time in the United States, African American filmmaking occasionally deployed documentary perspectives and created film collectives like the *Los Angeles School of Black Filmmaking* or *L.A. Rebellion* at UCLA (between 1969–1989), contributing to decolonial film movements. Urban ethnographies such as *The Single Parent Family: Images in Black* (Stormé Bright Sweet 1977) or *Cycles* by Zeinabu Irene Davis (1989), among others, created fictional narratives and documented everyday African American life from critical and feminist perspectives.

19 Rouch invited Oumarou Ganda to perform the ‘god’s voice’ (extra-diegetic documentary narrator) and explain the images to the viewer, also using sound artifacts such as traffic noise etc. The other (male) protagonists—with the exception of Dorothé, Petit Touré, Alassane Maiga, Amadou Demba, Seydou Guede, Kariodou Faoudou reenacted dialogues (Guéronnet & Lordon 1994; Comolli 2001).

20 For *Moi un Noir,* see the instructive and critical analysis by Hohenberger (1988); for a critique of Rouch’s filmmaking in general, see Ousmane Sembène (1997).

21 The model of the ethnographic film was transferred to his own environment. In *Chronique d’un été* (1961), Rouch and Edgar Morin worked with colleagues and friends to make a sociological study directly related to their own society in Paris, after the Shoah and the Second World War, in the era of decolonization and a modernizing society. They literally put people around a table and filmed the discussion, but also visited them in their homes and studios and on their way to the factory. The collaborative approach here lies in the moment of self-representation of one’s own life, mixing interviews, group discussions and ethnography. In this way, mixed forms were established by using spaces of self-expression alongside more sociological methods such as interviews.

22 In the framework of ethnofiction, recent collaborative filmmakers such as Gruber (2016: 33) have worked with acting in front of the camera as a means of shifting subject-object relationships.

23 Barclay (2003a; Barclay 2003b) distinguishes Fourth Cinema from films with Indigenous participation that do not benefit Indigenous people, such as the film *Whale Rider* (Niki Caro 2002), which is based on Māori stories and features Māori actors. Thanks to Adam Szymanski for this reference.

24 On the similarities and difference between Third (decolonial) and Fourth (Indigenous) Cinema, see Schiwy (2009: 55, 73–84, 163–164). Although the decolonial approaches of Third Cinema are also relevant for Fourth Cinema, the filmmakers of the former are more strongly rooted in Marxism and socialism.

25 Even before the rise of social media, film has been used for purposes similar to those of social media by distributing and communicating Indigenous perspectives to a wider audience.

26 Other more political examples can be found in the work of the Chiapas video collective.

27 “The act of video-making itself, when done to by an indigenous person or member of a local community, begins to ‘mediate’ a variety of social and political relationships within the indigenous community […]. In the process, cultural forms, together with the capacity and motivation of social actors to produce them, are reinforced, rearticulated, and transformed in various ways through the use of new techniques of representation and new social forms of utilizing and circulating them” (Turner 2002: 78).

28 This voice-over, which is still very much tailored to the *white* filmmaker Carelli, can certainly be criticized. The film is however highly informative, partly collaborative, and a piece of engaged filmmaking.

29 Within participatory research design, approaches often stem directly from participatory research practices that work directly with communities as experts of their own lives. The approach is used, for example, in social work and health care.

30 I only focused on collaborative practices in the field formerly dominated by anthropology. There are many other practices including, for example, creating feminist film collectives (see Holzberger 2020).

31 The materiality of knowledge production is just as important from a feminist perspective, since it reflects the division criticized by Barad and new materialism in general: that of matter as a passive being waiting to be shaped by ideas or forms, something that Gilbert Simondon criticized as hylomorphism (Simondon 2020: 2; for a feminist reading, see Grosz 2012).

32 The wave particle dualism has been discussed repeatedly in ethnography and regarding ethnographic filmmaking (see Harris in Perez 1998: 46; Schechner 1981: 10). Mostly, it was regarded as an epistemological problem, not an onto-epistemological one.

33 For decolonial methodology, see Smith (2013); on Indigenous epistemologies that challenge capitalist extraction, see also Lewis et al. (2018: 61).
